# Research ethics and refugee health: a review of reported considerations and applications in published refugee health literature, 2015-2018

**DOI:** 10.1186/s13031-020-00283-z

**Published:** 2020-06-20

**Authors:** Emma E. Seagle, Amanda J. Dam, Priti P. Shah, Jessica L. Webster, Drue H. Barrett, Leonard W. Ortmann, Nicole J. Cohen, Nina N. Marano

**Affiliations:** 1 Division of Global Migration and Quarantine, Centers for Disease Control and Prevention, 1600 Clifton Road, MS EO3, Atlanta, GA 30333 USA; 2 CDC/CSTE Applied Epidemiology Fellowship Program, Atlanta, Georgia USA; 3 Oak Ridge Institute for Science and Education, Oak Ridge, Tennessee USA; 4 Eagle Global Scientific, LLC, Atlanta, Georgia USA; 5 Office of Scientific Integrity, Office of Science, Centers for Disease Control and Prevention, Atlanta, GA USA

**Keywords:** Ethics, Research, Framework, Refugee, Health

## Abstract

**Introduction:**

Public health investigations, including research, in refugee populations are necessary to inform evidence-based interventions and care. The unique challenges refugees face (displacement, limited political protections, economic hardship) can make them especially vulnerable to harm, burden, or undue influence. Acute survival needs, fear of stigma or persecution, and history of trauma may present challenges to ensuring meaningful informed consent and establishing trust. We examined the recently published literature to understand the application of ethics principles in investigations involving refugees.

**Methods:**

We conducted a preliminary review of refugee health literature (research and non-research data collections) published from 2015 through 2018 available in PubMed. Article inclusion criteria were: participants were refugees, topic was health-related, and methods used primary data collection. Information regarding type of investigation, methods, and reported ethics considerations was abstracted.

**Results:**

We examined 288 articles. Results indicated 33% of investigations were conducted before resettlement, during the displacement period (68% of these were in refugee camps). Common topics included mental health (48%) and healthcare access (8%). The majority (87%) of investigations obtained consent. Incentives were provided less frequently (23%). Most authors discussed the ways in which community stakeholders were engaged (91%), yet few noted whether refugee representatives had an opportunity to review investigational protocols (8%). Cultural considerations were generally limited to gender and religious norms, and 13% mentioned providing some form of post-investigation support.

**Conclusions:**

Our analysis is a preliminary assessment of the application of ethics principles reported within the recently published refugee health literature. From this analysis, we have proposed a list of best practices, which include stakeholder engagement, respect for cultural norms, and post-study support. Investigations conducted among refugees require additional diligence to ensure respect for and welfare of the participants. Development of a refugee-specific ethics framework with ethics and refugee health experts that addresses the need for stakeholder involvement, appropriate incentive use, protocol review, and considerations of cultural practices may help guide future investigations in this population.

## Introduction

The UN Refugee Agency (UNHCR) estimates that, in 2018, there were over 25 million refugees worldwide, with nearly 37,000 people daily forced by conflict or persecution to flee their homes [1]. Complex migration patterns and poor access to healthcare often result in physical and mental health concerns throughout all stages of the refugee experience, including flight, asylum, and resettlement or return to their home country. Furthermore, refugees often have disparate risks of disease. These factors may contribute to health disparities among refugee populations and between refugees and non-refugees, which underscore the importance of conducting research specific to refugees and ensuring their representation in analyses (i.e., ensuring refugees are not excluded from analyses solely due to their vulnerability) [2]. High-quality data from such assessments are needed to develop evidence-based interventions and services and to guide national and international health policies.

The unique challenges refugees face can make them especially vulnerable to harm, burden, or undue influence in investigational settings [3–7]. For instance, refugees’ vulnerability is heightened by stress of relocation, family separation, exposure to violence or torture, uncertainty about the future, and prior or ongoing trauma [8]. Refugees also often lack the same rights as citizens in their countries of asylum, potentially resulting in limited political protections from maltreatment and limited access to healthcare, employment, or education [4]. Loss of assets during flight can also contribute to economic hardship and increase dependence on free social services for asylum-seekers. Some refugees are subject to continued oppression, restrictions on their liberty of movement, and gross power imbalances within refugee camps [6]. Additionally, low literacy (including health literacy) levels in some refugees can result in communication barriers and low understanding or misinterpretation of the investigation process [6, 9].

Ultimately, tension exists between the need for data to inform evidence-based practices and the need to protect vulnerable refugee populations from research-related risks [4]. The inherent dangers and extreme circumstances affecting people experiencing conflict and forced migration can make it difficult to simultaneously collect quality data and protect the rights of individuals with adherence to the highest ethical standards [7]. However, to exclude refugees from research or public health investigations because of their vulnerability violates the codes of justice and fairness [10], because evidence obtained from such investigations could inform targeted interventions, validate models of health service delivery, and ultimately protect the well-being of these individuals [3, 4, 7, 11]. Additionally, to only conduct research in non-refugee populations, even those with similar demographic characteristics, could provide an inaccurate and inadequate representation [3, 12, 13]. While there is clear justification for the need to conduct such investigations, it remains critical for investigators—as well as reviewers responsible for approving investigations—to address the myriad of complex ethical challenges present before, during, and after investigations [11].

Complex challenges are present throughout the refugee’s journey and may vary depending on whether the refugee is displaced (either internally or in a country of asylum) or has been formally resettled in a country that has granted permanent settlement. These challenges introduce complexities to conducting ethical investigations, including ensuring meaningful and voluntary consent, preventing real or perceived coercion to participate, minimizing undue influence (including economic), mitigating burden, and accounting for power imbalances between the investigators and participants [3, 5, 7]. For instance, participants could be motivated by the potential for economic gains or other tangible benefits (e.g., access to resources that are otherwise scarce), fear of consequences of not participating (e.g., when investigators in conflict areas are accompanied by armed guards, or fear of potential loss of benefits), or the possible misunderstanding that participation could help accelerate their resettlement process [7]. Additionally, refugee resettlement, for many countries, is a regulatory process involving relocation from an asylum country to another country that has granted permanent settlement, and typically includes mandatory health screenings [1]. Therefore introducing research or other public health investigations into the regulatory process of resettlement could blur the perception of voluntary participation. For instance, this could occur if there is confusion between mandatory medical screenings (or associated required disease treatments for resettlement) and voluntary screenings (or voluntarry treatments) for investigative purposes. Similarly, there is also the risk of confusing investigations, particularly those with a tangible intervention, with humanitarian aid. Respecting the rights, values, and beliefs of individuals and communities, as well as ensuring privacy (particularly in refugee camps), safety, and autonomy are also critical when engaging refugees. These principles become even more important given acute survival needs (e.g., food, shelter, clean water), risks of re-traumatization, and stigmatization of certain conditions or situations in the surrounding community (e.g., mental health issues, sexual assault) [3, 4, 6, 7]. Additional concerns arise in areas that may lack the capacity to provide appropriate technical guidance and oversight, such as in conflict settings [3].

The refugee context is ever-changing and therefore, the ethics principles followed and frameworks used to guide such investigations should be frequently examined and updated. Although a large body of literature exists outlining ethics principles and current debates in research ethics, there are few established refugee-specific ethics frameworks to guide such investigations, and no formal consensus about how basic research ethics principles should be interpreted in the refugee context. Additionally, while a handful of investigations have elicited data on thoughts and opinions about the investigation process and related ethical challenges directly from refugees [2], it is unclear how such responses and information have been translated to the activities of the wider scientific community [2, 14]. We conducted a preliminary assessment of recent published literature to understand the application of ethics principles reported in investigations involving refugees in recent years and propose considerations and potential best practices to protect the welfare of refugees in research or investigation contexts.

## Methods

### Information source, eligibility, and article selection

We reviewed reports of refugee health-related investigations published in English from January 2015 to September 2018 available in PubMed. Search terms were kept broad to increase the likelihood of identifying relevant publications. The search terms used were “refugee” or “refugees” in the article title to capture articles for which refugees were the primary focus. To keep the number of publications for review manageable, the PubMed search was restricted to these keywords within the title. Additionally, although refugee populations are similar to asylees (and asylum seekers), internally displaced persons, and other migrants, their circumstances, regulatory processes of resettlement, and types of protections can differ. Therefore, we limited our search to only refugees to narrow the scope and conduct a more focused review (however, the inclusion of these populations in addition to refugees did not lead to exclusion of an article). Given that similar ethical procedures are followed for both research and non-research data collections, and many publications did not distinguish between the two, both types of investigations were included.

Upon establishing this initial list in PubMed, we conducted the second stage of article selection by thoroughly reviewing the title and abstract (and in some instances where the abstract lacked the required information, the full article) according to the following inclusion criteria: (a) subjects of the article were refugees (not aid workers, clinicians, etc.); (b) primary topic was health-related; and (c) investigators directly interacted with the refugees included in the analysis (primary data sources). The last criterion consequently excluded investigations that solely used surveillance data, refugee databases, or chart reviews (secondary data sources). Review articles, systematic reviews, meta-analyses, notes from the field, letters to the editor, methodological papers, and policy papers were also excluded.

### Measures

After reviewing available literature on ethics in refugee investigations, the authors developed a data abstraction tool, with assistance from a refugee health expert and two public health ethics experts, to capture methodological aspects pertaining to ethics questions and dilemmas. We piloted the tool using 30 articles to be included in the analysis. As we reviewed those articles, we noted additional ethics-related scenarios raised or addressed, and we added a question to the data abstraction tool.

The final tool contained 64 variables (list of questions used can be found in ‘Additional File 1’), including both quantitative and qualitative questions. Information collected included characteristics of each investigation (year published, journal type, investigator’s home country, country in which the investigation was undertaken, type of institution, type of funding, investigation design, and primary health topic), as well as specific data points related to ethics considerations and methodological procedures. It was also understood that not all applications of ethics would be reported in the articles. However, due to the preliminary and exploratory nature of this analysis, we did not contact authors to obtain information not documented in the articles. If an author did not mention the information of interest, data abstractors filled in the variable as “not mentioned,” rather than “no” to avoid potential misrepresentation of the investigation or misclassification of the data (this was particularly pertinent to the post-investigation variables).

### Data collection

Four authors, abstracted data between July 1, 2018, and September 30, 2018. Each article was reviewed by one data abstractor.

After data abstraction was complete, 25% of each abstractor’s articles were randomly selected for a second review by one of the other reviewers to check for data irregularities or mistakes. The two abstractors discussed any differences and reached a consensus. For the 25% that underwent a second review, inter-reviewer agreement was high: average agreement rate per variable: 99.2%, standard deviation: 1.7%, range: 93.4 to 100%; three quarters of the variables had 100% agreement. For the remaining 75% of the articles, each record was scanned for any missing data points or inaccuracies (e.g., two answers contradicting each other).

### Data Analysis

Quantitative variables were analyzed using descriptive statistics. Qualitative variables were examined using a mixed methods approach including both an examination of the presence of a response (yes/mentioned vs. no/not mentioned) and an analysis of the response (identification of common themes).

## Results

### Article selection

**Fig. 1:**
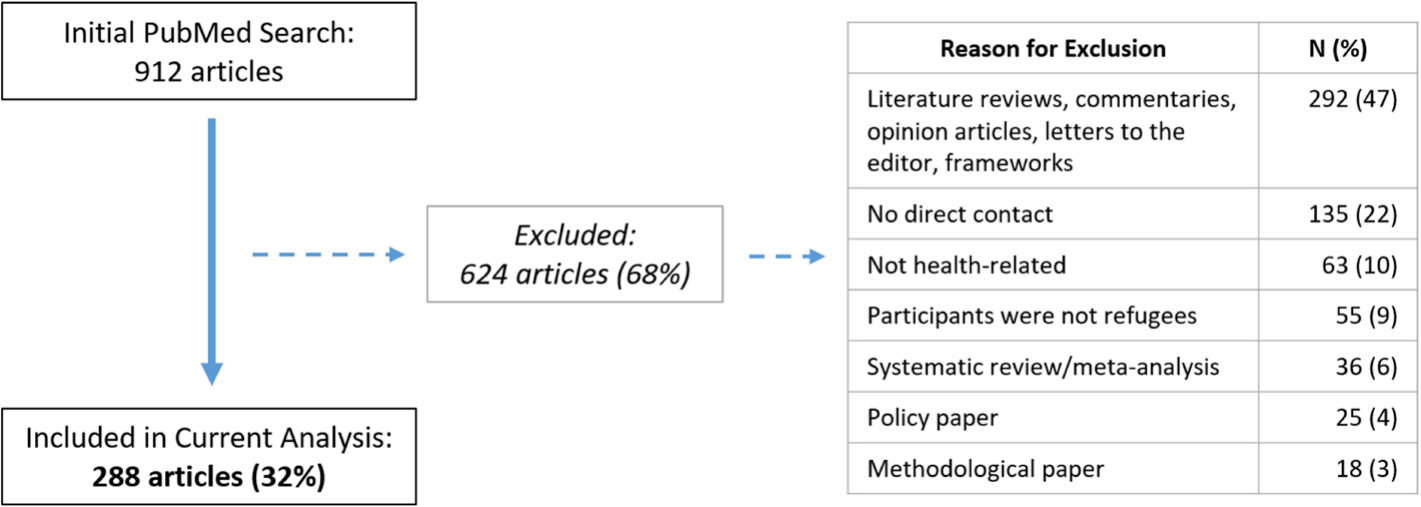
Article selection for a review of ethics considerations in published refugee health literature

Initial search results in PubMed yielded 912 articles, of which 288 (32%) were included in the analysis following the second stage of selection (list of articles included can be found in ‘Additional File 1’). Reasons for exclusion (*n* = 624) included: the article was a literature or systematic review, commentary, or meta-analysis (328/624, 53%); no interaction between investigators and participants (135/624, 22%); not health-related (63/624, 10%); refugees were not the subjects (55/624, 9%); and other (e.g., methodological, policy; 43/624, 7%) (Fig. 1).

### Investigation characteristics

Characteristics of the included investigations are shown in Tables 1 and 2 (and ‘Additional File 1’). Most of the investigators were from the United States (90, 31%), Australia (43, 15%), or Germany (16, 6%) with most investigations conducted by academic institutions (212, 74%). Funding was typically provided by a national governmental organization (76, 26%) or an academic institution (46, 16%). Approximately 33% (*n* = 96) of investigations were conducted during displacement (68% of these were in refugee camps), and most were cross-sectional by design (234, 81%). Nearly 42% used surveys or questionnaires for data collection; 33% used interviews, and 9% used focus groups, with similar distribution for both pre- and post-resettlement. Common topics included mental health (139, 48%) and healthcare access (22, 8%). Almost half (141, 49%) included at least one special subpopulation (e.g., LGBTQ, pregnant women, people with disabilities, children under 5 years old, adults over 65 years old).

**Table 1:** Characteristics of 288 articles in review of ethical considerations: author nationality, funding source, investigation design

Characteristic	***N*** (%)
Investigator’s Home Country, top four and other	
United States	90 (31.3)
Australia	43 (14.9)
Germany	16 (5.6)
Canada	15 (5.2)
Other	124 (43.1)
Primary Institution	
Academic institution	212 (73.6)
Medical institution	47 (16.3)
National government	17 (5.9)
Nonprofit/nongovernmental organization	7 (2.4)
State/local government	2 (0.7)
Other	3 (1.0)
Primary Funding Source	
National government	76 (26.4)
Academic institution	46 (16.0)
Nonprofit/nongovernmental organization	31 (10.8)
Medical institution	21 (7.3)
Private	14 (4.9)
State/local government	4 (1.4)
Other	13 (4.5)
None	11 (3.8)
Unknown/not mentioned	72 (25.0)
Investigation Design	
Cross-sectional	234 (81.3)
Prospective cohort	29 (10.1)
Randomized control trial	7 (2.4)
Other	18 (6.3)
Investigation Type	
Observational	249 (86.5)
Intervention	39 (13.5)
Time Point in Resettlement Process	
Displaced, refugee camp	64 (22.2)
Displaced, non-refugee camp (e.g., urban refugee)	31 (10.8)
Post-resettlement	192 (66.7)
Both, displaced and post-resettlement	1 (0.3)
Refugees Classified as Internally Displaced (among those displaced in camp or non-camp setting, *n* = 96)	7 (7.3)
Investigation Conducted in Conflict Zone (among displaced in camp or non-camp setting, *n* = 96)	3 (3.1)

**Table 2:** Characteristics of 288 articles in review of ethical considerations: health topic, data source, participant demographics

Characteristic	***N*** (%)
Primary Health Topic	
Mental health	139 (48.3)
Access to healthcare	22 (7.6)
General health profile	21 (7.3)
Maternal and child health	15 (5.2)
Nutrition and physical activity	15 (5.2)
Injury and violence	10 (3.5)
Other	66 (22.9)
Primary Data Source	
Surveys/questionnaires	120 (41.7)
Individual interviews	96 (33.3)
Focus groups	27 (9.4)
Human specimen samples	25 (8.7)
Medical records/programmatic databases^1^	12 (4.2)
Observations	5 (1.7)
Other	3 (1.0)
Age	
≥ 18 years old	160 (55.6)
< 18 years old	20 (6.9)
Both	92 (31.9)
Unknown/not mentioned	16 (5.6)
Special Populations *(not mutually exclusive, n* = 141*)*	
Over 65 years old	77 (26.7)
Under 5 years old	30 (10.4)
Pregnant women	17 (5.9)
LGBTQ	5 (1.7)
Disabled	2 (0.7)
Other	29 (10.1)
Sex	
Females	50 (17.4)
Males	7 (2.4)
Both	228 (79.2)
Unknown/not mentioned	3 (1.0)
Pre-resettlement Country, top four and multiple countries	
> 1 country	151 (52.4)
Syria	25 (8.7)
Burma/Myanmar	15 (5.2)
North Korea	12 (4.2)
Iraq	11 (3.8)
Other	74 (25.7)
Post-resettlement Country, top four and other (*n* = 193 that included refugees that has resettled after displacement period)	
United States	64 (33.2)
Australia	32 (16.6)
Germany	15 (7.8)
Canada	12 (6.2)
Other	70 (36.3)

^1^ Secondary data source used involved direct interaction with refugees in other parts of the investigation, qualifying the investigation for inclusion

### Ethics applications

#### Protocol review and consent

Ethics-related considerations and applications are presented in Tables 3 and 4. Approximately 94% of the included articles reported review of the investigation by an ethics committee. Two (0.7%) stated no review was conducted: one was an audit, and therefore, according to the authors, did not meet the criteria for an ethics review; one was a student’s thesis, and according to the authors, ethics approvals were neither required nor sought for student theses at their institution [15, 16]. For 15 (5.2%) articles, it was not stated whether an ethics committee reviewed. However, two of these 15 included language suggesting an ethics review (one discussed the protocol was reviewed by the “hospital administration,” which could include an ethics board; one discussed that a review was conducted in a prior investigation with the same sample population, but it was unknown if the prior review accounted for procedures conducted in the included investigation) [17, 18].

**Table 3:** Ethics considerations: ethics review, consent, risk minimization, privacy

Ethical Consideration	***N*** (%)
Ethics Review	
Yes	271 (94.1)
No^1^	2 (0.7)
Unknown/not mentioned^2^	15 (5.2)
Type of Investigators^3^	
≥ 1 internal investigator(s) (from country of investigation)^4^	264 (91.7)
Only external investigators (not from country of investigation)^5^	24 (8.3)
Location of Ethics Review, if all investigators from a different country (*n* = 23)	
External Review	7 (30.4)
Internal Review	4 (17.4)
Both	11 (47.8)
Unknown/not mentioned	1 (4.3)
Review of Protocol by Refugee(s)	
Yes	23 (8.0)
Unknown/not mentioned	265 (92)
Consent Obtained	
Yes	249 (86.5)
No^6^	2 (0.7)
Unknown/not mentioned	37 (12.8)
Format of Consent (*n* = 249)	
Written	116 (46.6)
Verbal	38 (15.3)
Both	27 (10.8)
Unknown/not mentioned	68 (27.3)
Reiterative Consent (*n* = 249)	
Yes	17 (6.8)
Unknown/not mentioned	232 (93.2)
Translation of Consent (*n* = 249)	
Yes	162 (65.1)
Unknown/not mentioned	87 (34.9)
Minimization of Risks, mentioned	
Mentioned	216 (75.0)
Unknown/not mentioned	72 (25.0)
Risk Minimization (*not mutually exclusive*, *n* = 216)	
Cultural sensitivity (interviewers’ demographics matched)	78 (36.1)
Provided trainings for investigators	61 (28.2)
Doctor/counselor present, or provided referrals	33 (15.3)
Piloted investigation, protocol reviewed by doctor, etc.	25 (11.6)
Other	109 (50.5)
Unknown/not mentioned	72 (25.0)
Privacy Measures Undertaken	
Mentioned	143 (49.7)
Unknown/not mentioned	145 (50.3)
Location of Data Collection	
Private	183 (63.5)
Public	24 (8.3)
Unknown/not mentioned	81 (28.1)
Data Stored as Deidentified	
Yes	55 (19.1)
Unknown/not mentioned	233 (80.9)
Collection of Identifiable Information	
Yes	66 (22.9)
No	47 (16.3)
Unknown/not mentioned	175 (60.8)
Use of an Interpreter	
Yes	191 (66.3)
Sometimes/when available	6 (2.1)
No	15 (5.2)
Unknown/not mentioned	76 (26.4)
Source of Interpreter (*n* = 197)	
Native speakers, from community	46 (23.4)
Native speakers, from another community	19 (9.6)
Native speakers, unspecified community	17 (8.6)
Non-native speakers	6 (3.0)
Unknown/not mentioned	109 (55.3)
Digital/Audio Recording of Any Portion of Data Collection	
Yes	78 (27.1)
No	57 (19.8)
Unknown/not mentioned	153 (53.1)

^1^ No ethics reviews: (1) audit that did not meet the criteria for an ethics review, (2) an ethics review was not required/sought for student thesis [15, 16]

^2^ Two of the unknown ethics reviews could have received an ethics review: (1) stated approval by hospital administration, (2) stated that prior study with same sample population had an ethics review [17, 18]

^3^ Using the list of authors

^4^ 248 (94%) mentioned an ethics review, 14 (5%) unknown, 2 (1%) no review

^5^ 23 (96%) mentioned an ethics review, 1 (4%) unknown

^6^ Two investigations stated “no” consent was obtained, citing routine screening/clinical care and quality improvement as justifications [19, 20]

**Table 4:** Ethical considerations: incentive use, establishing trust, transparency, benefits, results sharing

Ethical Consideration	N (%)
Use of Incentives	
Yes	66 (22.9)
Unknown/not mentioned	222 (77.1)
Investigator(s) Transparency^1^	
Mentioned	166 (57.6)
Unknown/not mentioned	122 (42.4)
Establishment of Trust by Investigators^2^	
Yes	114 (39.6)
Unknown/not mentioned	174 (60.4)
Stakeholder Engagement *(not mutually exclusive*, n = 262)^3^	
Health clinics/hospitals	110 (38.2)
Community members	88 (30.6)
Local nonprofit/nongovernmental organization	71 (24.7)
Community leaders/elders	43 (14.9)
National government	29 (10.1)
Local government	22 (7.6)
Local private business	6 (2.1)
Other	28 (9.7)
Unknown/not mentioned	26 (9.0)
Community Assisted with Recruitment	
Yes	145 (50.4)
Unknown/not mentioned	143 (49.6)
Cultural Practices Considered *(not mutually exclusive*, n = 121)	
Gender norms	49 (17.0)
Permission to conduct	22 (7.6)
Social hierarchy/order	23 (8.0)
Age hierarchy	9 (3.1)
Religious norms	10 (3.5)
Other	41 (14.2)
Unknown/not mentioned	167 (58.0)
Results Presented to Participants	
Yes	25 (8.7)
Unknown/not mentioned	263 (91.3)
Results Presented to Community	
Yes	9 (3.1)
Unknown/not mentioned	279 (96.9)
Social Justice/Health Equity Considered	
Mentioned	60 (20.8)
Unknown/not mentioned	228 (79.2)
Community Empowerment	
Trainings	21 (7.3)
Community education	20 (6.9)
Resources provided	12 (4.2)
Provided a voice	2 (0.7)
Unknown/not mentioned	233 (80.9)
Intervention Provided to Larger Community (*n* = 39)	
Yes	5 (12.8)
Unknown/not mentioned	34 (87.2)
Support of Intervention Post-investigation	
Yes	35 (12.2)
Unknown/not mentioned	253 (87.8)
Community Provided with Resources to Continue Intervention	
Yes	11 (3.8)
Unknown/not mentioned	277 (96.2)

^1^ Of those who mentioned transparency (not mutually exclusive): 58 (41.1%) held pre-investigation meetings, 54 (38.3%) ensured translation of consent/materials, 50 (35.5%) explicitly explained participation was voluntary

^2^ Of those who mentioned establishing trust (not mutually exclusive): 67 (58.8%) worked through community partners, 23 (20.2%) built relationships with community before investigation, 20 (17.5%) took actions to respect cultural norms

^3^ Type of stakeholder engagement: 144 (50.0%) recruitment, 36 (12.5%) data collection, 25 (8.7%) funding, 7 (2.4%) investigation design, 103 (35.8%) multiple of the previously mentioned types of engagement, 5 (1.7%) other forms of engagement

For 264 (92%) of the articles included, at least one of the investigators was from the country of the investigation. Of these 264, 248 (94%) mentioned an ethics review (1% none, 5% unknown). For 24 (8%) of all of the articles included, the investigation team was comprised of only investigators from outside the country of investigation. Of these 24, 23 (96%) mentioned an ethics review (4% unknown), and of these 23: the authors of 11 (48%) articles conducted both an internal (within the country of investigation) and external (within the investigators’ home countries) ethics review, the authors of 4 (17%) articles conducted an internal review only, and the authors of 7 (30%) articles conducted an external review only. Finally, of the 288 articles included, only 8% (*n* = 23) mentioned that their protocol was reviewed by representatives from the refugee community.

Obtaining consent was common (249, 87%). About two-thirds (162, 65%) of the investigations who stated they obtained informed consent mentioned translating the consent. Additionally, approximately 68% (197/288) mentioned the use of an interpreter at any point during the investigation. Only 7% (of the 249 who obtained consent) discussed making consent an iterative process (obtaining consent at each stage of the investigation to ensure continued understanding of research procedures, the voluntariness of participation, and an understanding of what individuals are consenting for at each stage). Two investigations stated no consent was obtained, citing routine screening or clinical care and quality improvement as justifications for not obtaining consent [19, 20]. Thirty-seven articles did not mention whether consent was obtained; of these, many were conducted as part of routine screening examinations at reception centers or during emergency or outbreak scenarios.

#### Risk minimization

Three-quarters of the investigations (*n* = 216) discussed methodological choices to minimize risks to participants (not mutually exclusive: 36% ensured cultural sensitivity; 28% trained the investigators before initiating the project; 15% ensured a doctor or counselor was available to participants in the event of physical or psychological distress; 12% conducted pilot investigations which allowed key community members to provide feedback on sensitive questions or best implementation strategies to ensure the safety, as well as mental and physical well-being, of refugees during the investigation). At least 20 articles stated that they engaged highly trained and qualified clinicians to administer the interventions and provided referrals as needed to culturally competent service providers [21–28].

#### Privacy

Overall, 50% (*n* = 1543) mentioned undertaking privacy measures, which included conducting the investigation on a sensitive topic in a school instead of a health clinic (i.e., a more neutral location) to prevent non-participants from associating the participants with the sensitive health topic and maintain the anonymity of the participants [27] and providing the opportunity to decline voice recording [29–31]. Private data collection locations were used in 64% (*n* = 183) of investigations (63% post-resettlement, 66% during displacement), including some in which participants were allowed to choose the location. Some investigations used homogenous focus groups based on sex or other demographic characteristics to create a safe space to protect participant identities [32, 33]; one investigation allowed individuals to opt for an individual interview over a focus group [34]. Of the 197 investigations with interpreters, 46 (23%) used native speakers from the community of interest. Finally, 19% (*n* = 55) of the articles stated that data were stored without identifiers, and many others indicated that data were stored securely using encrypted software, locked cabinet files, or secure servers.

#### Incentives (reimbursements)

Providing incentives, also referred to as reimbursements, was uncommon (66, 23%). Incentives were either non-monetary (11%), such as clothing or food, or monetary (89%). When monetary incentives were used, approximately one-fifth (20%) exceeded 30 USD, 61% were less than or equal to 30 USD, and 19% did not state the amount provided. Non-monetary or small monetary incentives (< 5 USD) were typically provided inside refugee camps, whereas larger monetary incentives were provided post-resettlement.

#### Transparency, trust, engagement, and respect

Approximately 57% of authors discussed measures undertaken to ensure transparency of the investigation within the community, including holding pre-investigation informational meetings; and 40% undertook measures to establish participant and community trust, such as working through trusted organizations or community leaders. Most authors (91% of all articles included, *n* = 262) discussed community stakeholder involvement, with common stakeholders being health clinics or hospitals, community members, and local nongovernmental organizations. Community members helped to recruit participants in 50% (*n* = 145) of the investigations. Cultural perspectives were considered in 42% (*n* = 121) of the investigations and included gender and religious norms such as matching the gender of the interviewer and/or interpreter to that of the participant, as well as ensuring homogenous focus groups (e.g., all female).

#### Post-investigation support

After the investigations, 9% (*n* = 25) of all of the 288 articles included presented results to participants, and 3% (*n* = 9) of all of the articles included presented results to the community. Approximately 13% mentioned post-investigation support to the participants or community (57% of these were post-resettlement investigations), and 13% of the 39 interventional investigations provided the intervention to the wider community after the investigation concluded. Discussion of community empowerment was more common for investigations conducted during the displacement period (30%) than after formal resettlement (14%). Twenty-one (7%) investigations empowered the community through trainings for community workers (healthcare workers, refugees hired and trained for the investigation, etc.), and 20 (7%) investigations provided health education to the community. Concerns of health equity and social justice were addressed by some (e.g., providing counseling or treatment when needed even when treatment was not part of the investigation [35], and providing vaccinations for all members in the community regardless of participation [36]). Given the explorative nature of most of the investigations included, sustainability of interventions was limited after project completion, with many investigators bringing screening equipment or other supplies for use only during data collection. However, in at least one case, resources were available to continue health programs or provide interventions to participants or the community beyond the investigation [37].

## Discussion

Although relatively limited in scope, our analysis provided an opportunity to describe the application of research ethics principles in refugee settings cited within recently published articles. With the growing numbers of displaced populations worldwide and the ever-changing context for causes of displacement, there continues to be a need for conducting public health research among refugee populations by diverse groups including governments, academic institutions, and non-governmental organizations. This assessment sets the stage for a more in-depth and comprehensive look at ethics concepts and their applications.

From our assessment, we found that the extent to which ethics principles were reported varied greatly across the refugee health literature we examined. Our findings highlight the need for a current understanding of ethics and the application of ethics principles in refugee health investigations. Moreover, there is still likely room for improvements to the investigation and review processes regarding ethics within the field of refugee health research. Additionally, the refugee context changes over time, as does critical thinking on research best practices, highlighting the need to repeat such analyses periodically to ensure research practices as they relate to refugees evolve accordingly. Although our current analysis provides important information regarding the present context and circumstances of refugees, the implementation of ethics principles in refugee health research should be revisited along with the changing landscape.

For the purposes of our discussion, we divided the investigational process into three phases—pre-investigation, investigation, and post-investigation—to discuss ethical challenges and potential best practices the investigator can undertake. However, many of these considerations span the full investigation process. We acknowledge best practices will vary by context, setting, and investigation characteristics. A summary of these proposed best practices described can be found in Table 5.

**Table 5:** List of some potential best practices^1^ to consider when conducting health-related investigation within refugee populations^2^

Pre-investigation Phase	Investigation Phase	Post-investigation Phase
- Ensure early engagement with key community leaders, stakeholders, and overall community (pre-investigation meetings, etc.) to ensure transparency and trust; and continue throughout investigation		
- Conduct a pilot investigation to allow key community members to provide feedback on sensitive questions and implementation strategies - Ensure review of protocol by an ethics committee in the countries of both the investigator(s) and the investigation, and by members of the refugee community, to minimize risks - Address potential power imbalances that may affect the investigation or who is represented - Prevent over-researching by searching literature before investigating - Train investigators (e.g., in cultural competency) - Differentiate investigation activities from social services - Inform community members on the purpose of the investigation - Give a voice to the community and key stakeholders to comment on the potential investigation and ask questions	- Ensure a private location for data collection (hard to find in refugee camps) - Carefully consider the risks to privacy when using interpreters from the community, and consider hiring interpreters from outside the community - Carefully consider the risks to privacy when conducting focus groups (consider separating by gender, age, or religion if appropriate) - If an incentive is used, place its value in context - Consider iterative consent - Minimize risks and harm (e.g., ensure a doctor/counselor is available in the event of physical or psychological distress) - Educate individuals on their rights as potential participants before they provide their consent - Ensure participation does not interfere with access to services - Ensure informed consent procedure is sensitive to cultural practices and norms, and practical for populations that have low literacy or little understanding of the investigation process - Present preliminary results to stakeholders to improve interpretation of results	- Present final results to both participants and their community - If an intervention is provided engage with the community and stakeholders to ensure its sustainability - Provide community members with job skills to be used post-investigation - Empower community health workers through trainings - Provide continued and sustainable health educational classes for the community - Allow participants and community members to comment on the results - Identify ways to provide immediate benefits in addition to long-term, sustainable ones

^1^ Best practices, and the weight awarded to each practice, should and will vary by context, setting, and investigation characteristics; not an exhaustive list

^2^ And to consider discussing these considerations in published literature, as they are able

### Pre-investigation phase

To ensure investigations are conducted with respect for and protection of the participating individuals and community from the onset, ethical considerations should be at the forefront throughout the pre-investigation phase, during which questions are refined and methodological details are decided (e.g., location of investigation, content and logistics of consent, appropriate ethical and community approvals, mechanisms for recruitment of participants).

Early community and stakeholder engagement is critical during the pre-investigation phase. Such engagement can help to bridge cultural differences and establish trust within the community, as well as provide an outlet for the community to voice their concerns and thoughts [9, 38]. For instance, this engagement can take the form of a formal community needs assessment or informal community-wide meetings to identify the type(s) of services needed or the benefits and risks of a specific project or intervention. Pre-investigation meetings also enable investigators to fully communicate to community members how and why an investigation is being conducted, and the potential benefits for the community. Feedback from the community through key informant interviews or focus groups can also shape investigation protocols and procedures to add cultural competence. For example, the content and format of consent can meet participants’ information needs, or location of interviews can show respect for the community [9]. Transparency during this phase can address concerns regarding the impact on the community and answer questions for those unfamiliar with the investigational process [6]. Transparency and open communication with the community also ensure the investigation addresses information gaps in addition to the priorities and interests of the investigator [11].

Furthermore, in conflict settings where investigators need security guards, community engagement is particularly important to address potential power imbalances. For instance, in some cases, armed guards or other authority figures may accompany investigators to ensure their safety. Such non-routine security measures can unintentionally produce undue fear and the perception of coercion, particularly among refugees previously mistreated by authority figures. Therefore, prior community engagement may be needed to establish trust and ameliorate these feelings [5, 39].

Additionally, during community engagement and project development, investigators should (a) clearly differentiate investigational activities from provision of social services or humanitarian aid, or processes required for resettlement and (b) ensure that participation does not affect provision of services (e.g., by scheduling interviews outside ration distribution hours, or ensuring the investigation does not take medical personnel away from routine duties) [6, 11, 13]. Investigations differ from service provision in that the success of an intervention used in an investigation is still unproven. Additionally, often only a small portion of the eligible population is recruited to participate in an investigation. Yet for those unfamiliar with investigations, these distinctions can be confusing and appear unfair, as when only a small portion of the population receives investigational hygiene products or nutritional supplements. It is also essential that participant recruitment and selection strategies are unbiased to ensure all eligible individuals or groups have an equal opportunity to participate. When some people are excluded, transparency is needed to adequately explain this differentiation to the entire community, and to convey that the goal of the analysis is to provide a benefit for everyone in the future [11, 40].

Overall best practices for community engagement include pre-investigation meetings with community leaders and members [6]. Such leaders can often be identified through UNHCR and nongovernmental organizations in the refugee camp or community where the investigation is to be held. However, investigators must anticipate that the power hierarchy within the refugee population might unduly influence potential participants or predetermine which individuals or groups have access to the research, and take steps to minimize such influences [7, 41]. It is also important to note that although community engagement is initiated pre-investigation, significant benefit comes from its continuation throughout the investigation.

Another key step during the pre-investigation phase is an ethics review of the investigation protocol. It was promising to see that the majority of investigations mentioned review by a formal ethics committee; however, not all discussed completion of this review in both the investigators’ home country and the country where the investigation took place. Some investigators’ ethnic and cultural backgrounds differed from those of the participants, setting the stage for potential clashes between cultural, gender, or religious norms [42]. An ethics review within the country of investigation helps to ensure methodologies align with community practices. It also minimizes risks not often identified by an ethics committee unfamiliar with the local culture [38, 43].

However, even ethics committee members inside the country of investigation may be unfamiliar with refugee needs, circumstances, and vulnerabilities and therefore not fully able to represent the interests of refugees displaced from another country. Thus additional review by members of the refugee community in which the investigation is occurring may prove most insightful. Few articles mentioned the presence of a refugee on the committee or review of the protocol by refugees. Such reviewers are often better positioned to weigh the risks and judge the value of the investigation and determine the cultural feasibility and appropriateness of the methods [13]. These reviewers may also play the role of ethics committees in locations that lack official ethics committees, such as conflict settings.

Finally, investigators should carefully consider the need for and utility of their investigation before initiation to prevent overburdening refugees with similar or repetitive investigations, often referred to as “over-researching” [3, 6, 7, 38]. In our review, we were unable to assess the degree to which investigators strove to prevent over-researching. The risks of over-researching are particularly relevant in mental health investigations, which may pose a risk of re-traumatization. All investigations should have both practical and actionable outcomes that would not be possible without the investigation [6, 13]. Best practices to prevent over-researching might include thorough literature reviews and consultations with experts in the subject area to understand whether a proposed investigation is truly necessary to develop effective interventions. Another could be stricter requirements by ethics review committees that investigators demonstrate that their proposed investigations are truly warranted and meet the “reasonable person standard,” which requires investigators to assess the need for a particular investigation to avoid duplication and excess burden [44, 45]. The timing of the investigation may also affect the degree of burden on the target population. For instance, resettled refugees may feel less burdened by investigations because many of their acute needs have been addressed, while those not yet resettled may be more vulnerable. Overall, the investigation’s impact should be carefully considered to ensure the findings can effectively benefit the population; and especially in emergency response settings, new investigations should be initiated only when necessary data cannot be obtained in other ways [11].

### Investigation phase

Ensuring ethical implementation during the investigation encompasses the overarching principles of minimizing stigma and other harm, ensuring privacy and respect, and preventing undue influence or coercion. A majority of the investigations in our analysis ensured a private location for data collection, with some investigations only collecting de-identified data or choosing individual interviews over focus groups (although held in private locations, the latter are not truly private) [7, 38]. Nonetheless, finding a private location can be challenging in refugee camps, and even if the information shared by participants is kept private, it may be difficult to keep participants’ names confidential. Participation may involve disclosing characteristics that could increase risks of stigma or place an individual in danger if revealed to other community members (e.g., rape, sexual identity, mental illness, sexually transmitted infections) [5, 7]. Additionally, many of the articles included in our analysis mentioned the use of interpreters from the community, which can also affect privacy. Although at the surface level these individuals appear to be the most appropriate cultural brokers, they can also (a) bias the results in that, fearing stigma, the participant declines to share information, or, potentially worse, (b) spread sensitive and private information disclosed by the participant into the community [5, 38, 39]. To mitigate these risks, one could seek community input to assess the acceptability of using interpreters (which could vary by health topic and setting) from the community, and if deemed unacceptable, hire bilingual individuals from outside the community when possible. Ultimately, strict privacy and confidentiality measures help to prevent further emotional or physical harm. As in the pre-investigation phase, cultural practices should be considered when choosing methods for ensuring privacy and confidentiality [38]. If focus groups are considered necessary for sensitive investigations, optimal standards could include separating the groups by gender, age, religion, or other factors, if deemed appropriate [9, 13, 38, 39].

Investigators also need to be knowledgeable about the refugee experience to prevent undue influence [5]. For instance, in our review, incentive type and value ranged greatly. Use of incentives needs to be placed in context to understand the amount of influence they may have. Five USD inside a refugee camp in Uganda, for instance, can have a drastically higher value than the same incentive provided after resettlement in the United States. Additionally, if implemented inappropriately, incentives can contribute to inequality between participants and non-participants. Engagement with community leaders, local nongovernmental organizations, or other refugee health experts may help in establishing whether incentives have appropriate values or whether they should be offered at all.

The complexities surrounding meaningful informed consent also require attention [5, 38]. Many refugees have never participated in investigations before and may not understand the concept of research or the investigation process [13]. For example, in a previous assessment of refugee knowledge around scientific investigations, less than half (44%) of refugee respondents correctly answered “false” when asked whether “once somebody starts participating they are not allowed to quit” [46]. Although this percentage may have been influenced by communication barriers, it indicates that informed consent may not always be communicated effectively to refugee participants.

Our review of the literature reveals promising adherence to basic ethics principles for obtaining consent, a cornerstone to protecting participants’ autonomy in all public health and biomedical investigations [47]. However, one should also consider the format and type of consent to ensure complete understanding and respect. Most of the investigations we reviewed obtained written consent, but not all addressed language barriers, literacy levels, languages that do not have a true written form, and cultural normalcy (e.g., in some cultures, verbal consent holds a higher value) [13]. Translation of consent into the language the participant prefers may minimize misinterpretations. Additionally, although written consent remains the norm, it can prove challenging in refugee investigations. Options for non-written consent may include verbal consent, audio or video-recorded consent, or witnessed consent (a witness signs) [48]. Innovative methods such as videos, illustrations, or other visual aids to help explain and obtain consent may also prove useful [48]. Few articles mentioned the use of iterative consent, a process by which individuals consent at each phase of the investigation. Such iterations remind refugees about the research procedures and that their participation is voluntary, which can help reduce feelings of pressure to complete the investigation [5]. Additionally, in the event that human biologic specimens are collected, ownership of these specimens should be discussed with respect for cultural and religious beliefs about the human body during the consent process. Ultimately, informed consent must be sensitive to cultural practices and norms, as well as practical for populations that have low literacy rates or limited understanding of the research and investigation process to help ensure true autonomy [3, 6].

### Post-investigation phase

Finally, although often overlooked, the application of ethical practices should not cease after the investigation has ended. Few articles discussed ethical considerations for this phase of investigations, but such considerations are not always reported in published articles and can occur after publication. Nonetheless, post-investigation activities include aspects of social justice and health equity that should not be ignored [40, 49]. Best practices post-investigation may include empowering community health workers through trainings during or after the investigation; employing local refugees to help with the investigation, thereby providing them with job skills that will be useful post-investigation (this would often happen in the pre-investigation stage with the benefits extending into post-investigation); providing the means to sustain health education classes for the community; and providing resources to continue an effective intervention (or if applicable, providing the intervention to the wider community) [6, 40, 50]. Given the transient nature of refugee populations, the timing of such reciprocity is key. Sometimes participants move before investigation results are translated into permanent interventions; therefore, investigators should identify ways to provide immediate benefits to participants who may not see the investigation’s long-term impact [7, 49]. Lastly, it is important to consider ensuring “reasonable availability” of an intervention that is demonstrated to be effective (i.e., ensuring the intervention is available free or at a reasonable cost to the community in which it was tested) [50].

Transparency after the analysis is also key and includes presenting the findings to both the participants and the wider refugee community [50]. Power imbalances can be addressed here by allowing individuals to comment on the results and their interpretation rather than merely hear the investigators’ final framework or conclusions [6]. This, as well as other best practices outlined above, follow the community-based participatory research approach (an approach that collaboratively includes all parties involved throughout the research process, acknowledging that each party brings unique strengths and perspectives) [51].

Ultimately, many of the basic principles discussed above should also be applied in non-refugee public health investigations. Nonetheless, refugees as a population have unique vulnerabilities that warrant a greater level of diligence in avoiding research-related harm. For instance, refugees often differ from non-refugees in the degree of political protections or types of social services offered to them. Therefore, the manner in which these principles are applied, and the emphasis given to certain principles, should be specific to the refugees’ individual circumstances. The addition of requirements by funding agencies and journals to outline and document procedures used may also help to ensure adherence to ethical guidelines.

### Limitations

Our analysis was subject to several limitations. Given that not all journals require publication of all ethics applications we assessed, and publication requirements (word count, structure, reporting of post-investigation activities, etc.) vary, ethics considerations may have been omitted from the published manuscripts. As our analysis only accounted for actions reported within the published literature, we acknowledge our results are likely an underestimation, particularly of post-investigation activities. Therefore, we have drawn conclusions only in regard to the procedures and protocols investigators reported, and argue for the importance of increased reporting of ethics applications. It is also important to acknowledge that differing approval processes between research and non-research investigations may have affected the application of ethical standards. Additionally, the use of only one publication database and the inclusion of only articles in English language may have limited the types of articles included (e.g., social science articles may have been excluded). Furthermore, restricting the time of publication to 2015 through 2018 prevented the ability to analyze how adherence to reported ethical protocols evolved or varied over time. The analysis was also restricted to those with the words “refugee” or “refugees” in the title, which may have excluded some articles that did not mention the study population in the title but would have otherwise met the inclusion criteria. Additionally, this analysis was specific to refugees and the search criteria excluded other migrant study populations, such as asylees (when the article was solely focused on non-refugee migrants; some articles included both refugees and other migrants, and these papers were included in the current analysis). Although many of the basic ethics principles examined in our analysis translate to other migrant populations, there are also differences in the regulatory processes of resettlement that makes these groups different and warrant a deeper assessment of ethics principles for different migrant groups beyond the scope of this analysis. Future in-depth assessments should focus on these populations. Data collection errors might have been reduced if resources had been available to conduct a second review of all articles. However, given the high agreement rate on articles for which we did a second review, the number of data collection errors is likely low. Finally, due to the narrow scope of our analysis, we limited inclusion to primary data collections. Nonetheless, ethical dilemmas can arise in secondary data analyses. For instance, many government agencies and nongovernmental organizations collect program data intended for internal use that are later used by investigators. Such scenarios should be explored in future reviews.

### Recommendations

Refugee investigations are needed. Investigators, in addition to maintaining the highest level of scientific integrity and rigor, have a duty to act ethically. The investigators’ responsibility extends beyond securing an ethics committee approval, especially given that refugees are not always included as a “vulnerable population” (populations identified in need of enhanced protections), to ensuring protection of the welfare and dignity of participants (and documentation of that protection) [11]. For instance, the US human subjects research regulations (federal policy for protection of human research subjects, which defines the processes for ethics review and approval) does not include refugees as a vulnerable population [11]. Yet the field of ethics is dynamic, and often difficult to navigate. Ethics is far from an exact science, and emphasis on specific considerations and applications varies with population and setting. Our objective was to describe the breadth of application of ethics principles and identify possible gaps in their implementation, potentially stemming from the lack of a guiding research ethics framework in the field of refugee health.

There exists a need for an updated and comprehensive refugee-specific ethics framework to guide future investigations. This framework should include the basic principles found in both the Belmont Report and current public health frameworks, as well as other philosophies, including ethical guidance regarding underserved communities and ethics of community practice [11, 49, 52–54]. It would also require a degree of specificity to refugee populations that accounts for their heightened vulnerability and the characteristics that contribute to this vulnerability that make refugees unlike other individuals (e.g., limited political protections of stateless individuals, living conditions inside refugee camps, prior torture or trauma, economic instability, population mobility). Such a framework requires flexibility in order to remain applicable to the diversity in age, socioeconomic status, education level, and cultural practices among refugee populations, meaning that the weight given to a particular principle can and often should vary by context [49]. For instance, privacy should be held to the highest of standards when interviewing LGBTQ youth in a refugee camp, where disclosure of sexual orientation could place the individual in danger; but privacy may not take priority when examining less-stigmatized topics [49]. An established framework that identifies innovative solutions to protect refugees while ensuring scientific validity would aid in improving future investigations. Furthermore, the principles outlined in the framework could provide guidance and context to assist ethics committee reviewers in assessing the ethical integrity of proposals, particularly for reviewers unfamiliar with refugee health.

The best practices we identified both in our literature review and in developing the methodology for our literature review may help lead to developing a framework. To provide a supporting structure for such a framework, we propose three key areas: *engage, educate, and empower*.

### Engage

Engage with stakeholders, community leaders, and community members before and throughout the investigation. This engagement helps to create trust, transparency, and collaboration. Additionally, early engagement with ethics committees helps to minimize risks of adverse outcomes.

### Educate

Educate investigators (interviewers, data collectors, analysts, etc.) on topics such as cultural competency and ethics. Educate community members on their rights as potential participants in the investigation before you ask for their consent. Ethics committee reviewers may also benefit from education to ensure they are aware of the complex vulnerabilities of refugee populations.

### Empower

Empower participants by ensuring they understand that participation is voluntary. Empower the community post-investigation by presenting results at forums (where community members can comment), ensuring sustainability of an intervention that the community can assume ownership of, or providing classes or trainings.

## Conclusion

Overall, we found that ethics information is not always systematically reported in current published literature reporting on investigations involving refugees. Publications often did not document ethical considerations, and therefore, we encountered difficulties discerning which principles were followed. We did find two key points: (1) review of the investigation protocols by refugees themselves was reported infrequently (only 8%), and (2) post-investigation support or engagement for both the participants and their community was reported minimally (although the authors recognize the potential for omission of these in manuscripts submitted for publication, if not main focus of the investigation).

In conclusion, our analysis identified a number of complex ethical challenges in conducting refugee health-related investigations and found evidence of room for improvement in adherence to ethics principles and their documentation in resulting publications. Most importantly, we have described the unique characteristics of refugee populations that suggest a need for greater emphasis on particular ethics principles and warrant the development of a refugee-specific ethics framework to aid investigators in the field. Scientifically valid investigations with ethically collected data provide the foundation for policy and interventions, and therefore, investigators should make the fullest effort to ensure respect and safety for refugee participants and their communities.

## Supplementary information

**Additional file 1.** Articles included in review and additional characteristics. Additional file 1’ includes the list of the 288 articles used in the review of ethical considerations in refugee health literature, additional characteristics of these 288 articles, and a list of the 64 variables abstracted from each article.

## Data Availability

The datasets used and/or analyzed during the current study are available from the corresponding author on reasonable request.

## Abbreviations

UNHCR: UN Refugee Agency
LGBTQ: Lesbian, gay, bisexual, transgender, queer
USD: United States dollar
