# Supplementary material for: Research ethics and refugee health: a review of reported considerations and applications in published refugee health literature, 2015-2018
Source: Confl Health. 2020 Jun 20;14:39. doi: 10.1186/s13031-020-00283-z (PMC7305588; doi:10.1186/s13031-020-00283-z)
Supplement: Supplementary file 1 — Additional file 1. Articles included in review and additional characteristics. Additional file 1’ includes the list of the 288 articles used in the review of ethical considerations in refugee health literature, additional characteristics of these 288 articles, and a list of the 64 variables abstracted from each article. [file 13031_2020_283_MOESM1_ESM.docx]

**­Reviewer Responses**

**Article Title:** “Research Ethics and Refugee Health: A Review of Reported Considerations and Applications in Published Refugee Health Literature, 2015-2018”

**Manuscript Number:** CONF-D-19-00147

**EDITOR COMMENTS:**

**Editor Comment #1:** Thank you for this submission. This is a very good article and we look forward to being able to accept it after some small revisions. The Conclusion contains excellent material, but I agree with Reviewer #1 that it is very long. Having it as a separate Recommendations section would be preferable, and then leaving a short Conclusion section.
The other suggestions are relatively minor. Best wishes, Guest Editor.

***Author’s Response:***
Thank you for this comment. We agree that the conclusion section is very long and have taken the reviewer’s advice to move the “recommendations” originally included inside the conclusion section to a new subheading called “Recommendations” under the “Discussion” section of the paper. We appreciate this comment as it greatly improves the flow of the discussion/conclusion sections. These changes are reflected in the text in lines 580-691 (pages 26-31).

**REVIEWER 1:**

This is a well written paper on an important topic. Please see my minor comments below:

**Reviewer Comment #1:** Page 7, lines 140-146, this is a very long sentence, recommend it be split into 2.

***Author’s Response:***

Thank you for this comment. We agree that this sentence is long and therefore difficult to understand. Therefore we have split the sentence into three sentences that are more manageable for the reader to follow and comprehend. Specifically, we eliminated the large amount of text in parenthesis and put this information in its own sentence. These changes have been made in lines 140-149 (page 7).

**Reviewer Comment #2:** Page 8, line 173, "asylees" - perhaps this was meant to be asylum seekers?

***Author’s Response:***

This is an important distinction to make. The terms “asylee” and “asylum seeker” are somewhat synonymous. An “asylee” is someone who came to another country without official refugee status, who has since been granted legal status by the government. An asylum seeker is anyone who has fled persecution in his/her home country and is seeking safe haven in a different country but has not yet received any legal recognition or status. Therefore the use of both terms would be most appropriate here when we discuss inclusion/exclusion criteria because neither were included in our review. Nonetheless, both groups are similar to refugee populations and therefore the exclusion of both groups should be mentioned. The addition of “asylum seekers” as an excluded group was added to the text on line 178 (page 8).

**Reviewer Comment #3:** Page 13, line 287, it is noted under the heading 'Privacy', that some studies conducted "the investigation in a school to disguise the purpose of the activity" This reads as if this was a study which used deception to mislead research participants? Is that correct? I would recommend clarifying what is meant by "disguise"

***Author’s Response:***
Thank you for your assistance in helping us to clarify this point in the text. The study was not using deception to mislead the research participants, but rather the investigators used deception to mislead non-participants. Given the sensitive topic of the study, the researchers wanted to protect the identifies of the participants. Therefore, they held the study in a location that was a non-assuming place (i.e., not a health clinic) so that non-participants would be less likely to associate the participants they saw go into the school with the sensitive health topic (for instance, they could just be picking up their children from school). We have edited the text describing this to ensure the correct meaning is portrayed in lines 293-295 (page 13).

**Reviewer Comment #4:** Page 25, Limitations, recommend including that only papers in English language were included

***Author’s Response:***

We agree with the reviewer that this is a very important limitation to also include in the text. This limitation had been added to the manuscript on line 559 (page 25).

**Reviewer Comment #5:** The Conclusion section is rather long, this should really just be a paragraph or two summing up the most important messages from the manuscript. I would recommend the information on recommendations and future research be included in a "Recommendations" sub-heading within the Discussion instead. Also, the information on development of a refugee-specific ethics framework (page 26-28) should really be included within the Discussion section, perhaps under a sub-heading.

***Author’s Response:***

Thank you for this comment. We agree that the conclusion section is very long and have taken the reviewer’s advice to move the “recommendations” originally included inside the conclusion section to a new subheading called “Recommendations” under the “Discussion” section of the paper. We appreciate this comment as it greatly improves the flow of the discussion/conclusion sections. These changes are reflected in the text in lines 580-691 (pages 26-31).

**REVIEWER 2:**

This is an excellent manuscript that addresses a very important and relevant topic. It is very well organized and written. It addresses important issues and provides a very important and useful set of recommendations based on the results of the review.

**Reviewer Comment #1:** While the manuscript is very well written, it did note a couple of small typos. On p. 12, line 256, there should not be a bracket after "investigation".

***Author’s Response:***

Thank you for this edit. We have removed the unnecessary bracket on line 261 (page 12).

**Reviewer Comment #2:** On p. 12, lines 258-261, the way "conducted" is used here is not appropriate. It states that the articles conducted various reviews. It should state that the authors of the articles conducted various reviews, or that the articles reported on teams that conducted reviews.

***Author’s Response:***

Thank you for your suggestion on how to clarify this sentence. We agree with the reviewer that the wording within this sentence should be changed to “the authors of the articles.” This change is reflected in the text in lines 264-267 (page 12).

**Reviewer Comment #3:** There may be some others, so please take this chance to proofread the manuscript again. Otherwise, this is a very nice piece of research.

***Author’s Response:***

Thank you for this suggestion to proofread the article again. We have completed a thorough proofreading prior to resubmission. Four minor grammatical changes were made during this proofreading process, which include: (1) a comma added to line 347 (page 16) after “academic institutions,” (2) the word “the” added to line 390 (page 18) before “perception of coercion,” (3) the word “where” was replaced with the word “when” on line 471 (page 21) before “from outside the community,” and (4) the comma after “human body” was removed on line 510 (page 22).
